# Address of Ohio College of Dental Surgery

**Published:** 1880-04

**Authors:** M. S. Dean


### THE DENTAL REGISTER.

Vol. XXXIV.] APRIL, 1880. [No. 4.

### ADDRESS

DELIVERED AT THE COMMENCEMENT OF THE

### Ohio College of Dental Surgery,

March 6th, 1880.

BY M. S. DEAN.

*Mr. Chairman, Friends, and Gentlemen of the Graduating Class:*

Eugene Sue tells us that the Piannakotaws—a tribe of South American Indians—kept up a standing army of invincible warriors; that commissions in this army of braves could not be bought; but that aspirants to this noble profession were accepted only after having submitted to the most searching examination, and the severest tests of their courage and endurance.

During this examination (or trial) which occupied *nine* successive days and nights, the applicant abstained from food and sleep. But I need not dwell upon the details of the mode of examination ; suffice it to say, that the new recruit was subjected to all the cruel tortures that savage ingenuity could devise.

On the ninth day, if the applicant still survived, and showed no signs of weakness or discomfort, his courage and endurance were regarded as satisfactory; and the exercises were concluded with the delivery, by one of the Nestors of the tribe, of a long harangue, laden with much good counsel, and many well-worn truths. Now whether the address of this "Pylian Sage" of the forest, was regarded as a *culminating* test of the patient endurance of the noble child of Mars, M. Sue fails to inform us; but we may logically infer that it was.

Gentlemen, while rumaging over my mental storehouse, in search of something appropriate for this occasion, I have been led to wonder if, in admitting new recruits into our profession, we have not borrowed some of the cruel customs of the Piannakotaws. Not that I discover in your preparation and examination here any resemblance to that practiced by this tribe of braves; nor that I see any very striking similarity between the two noble professions. But the concluding ceremonies are so evidently identical, that I am led strongly to suspect that our idea of a closing address was borrowed from them.

In acquiring your degree of Doctor of Dental Surgery you have undoubtedly endured some hardships, and possibly some privations; but these are trifling as compared with the torture it is in my power to inflict upon you this evening. Addresses of welcome, of congratulation, and the like, are very generally dreaded, and, if possible, evaded, by those for whom they are prepared. Henry the Fourth, of France, dodged them, as he would the teeth of a dangerous dog; and he attributed his prematurely grey hairs to the speeches to which he had been compelled to listen. In the whole history of the world only one man has been found—*speech proof.*

In view of these facts, though I fully appreciate this opportunity, and enjoy the temporary power that has been vested in me, yet I have determined to be merciful to you; and I shall endeavor not to overload my address with too much weighty advice, nor with too many ponderous truths.

Gentlemen graduates, you have earned and secured all the honors that this institution of learning can confer, and you have received all that special instruction which is adjudged necessary to qualify you to commence the practice of dentistry. If all who have entered the profession before you, had enjoyed similar educational advantages, you would have found the profession a more respected and honored, as well as a more learned calling. But while you enter it under far more favorable auspices than did most of us, you find it also, in an educational point of view, a far different profession from what the most of us found it. It has been steadily advancing in knowledge and usefulness from year to year, notwithstanding the sad deficiency of the great mass of its members in a preliminary professional education. But this does not prove that a systematic collegiate course of instruction is not essential to its prosperity, for it has been mainly through the means of our dental colleges, and the graduates who have issued from them, that the less educated members have caught the spirit of study and investigation.

Fortunately for you gentlemen, our profession has not yet attained its full growth, nor is it in all its parts equally vigorous or equally advanced. While some of its branches are already bearing excellent fruit, some of them are as yet only in bloom, and others barely in bud; and there are still others which will require most scientific culture, before they will even give token of either fruit or bud. Fortunate for you, I say, that this is so; for if it were otherwise, your activities and energies would become dwarfed, and you, like the languishing Alexander, who wept because there was no other world for him to conquer, would soon become feeble and childish from mental stagnation. But you need not weep, you have more than worlds to conquer. Many fields of investigation lie open to the scientific dentist. Your intellects will not become dwarfed or cramped for want of room to exercise; the farther you pursue your researches, the broader and more inviting will the prospect open before you. Much of this vast territory has been partially explored; but much of it is still the *terra ingognita* of our profession. Here each of you will find ample scope for your individual tastes and your natural and acquired abilities. If your original investigations are to be carried on with a view of benefiting the profession as well as yourselves, you must concentrate your forces upon some one branch or division of a subject—such for instance as Embryology, Histology, Physiology, Therapeutics, Pathology, Chemistry, &c. But you must possess a *general knowledge* of an entire subject before you commence your *special* researches upon any of its branches, else you will arrive at erroneous, quite as often as correct and valuable conclusions.

The lack of this general knowledge, which is too often exhibited by investigators, sometimes subjects us to the ridicule of more thoroughly scientific bodies, and lessens their confidence in our just claims to accurate investigation. Before commencing to investigate for yourselves any special department of science, you should gain from our best literature a broad and comprehensive view of the whole subject. In this way you may often acquire in a few hours, facts and principles which your *unaided efforts* would not discover in a life time. In fact, our science will scarcely gain one inch of ground by the *independent* and *isolated* labors of individuals; their investigations must be aided by the accumulated and the accumulating experience of those who have labored, and of those who are now laboring in the same direction. I think it not necessary to elaborate this point.

You will find that among many of the more progressive members of our profession who commenced the practice of dentistry without first securing the degree of Doctor of Dental Surgery, many have, after years of study and practice, sought the "sweet food of sweetly uttered knowledge," which is found in the lectures delivered in our Dental Colleges. And again many others who have added years of study and practical knowledge to that acquired in securing their degree of D. D. S. are pursuing full courses of medical instruction, to slake their thirst for knowledge by "drinking largely" of these "Pierian Springs." Among such men you will find congenial companions and worthy co-laborers.

You will, probably, at least during the first years of your practice, find your profession admirably adapted to study and experimentation with ample opportunity to pursue your studies without fear of interruption. The most of us, your seniors, found it "a profession of genteel leisure" for several years, and indeed I am afraid some of us find it so still.

These leisure hours may, however, be spent profitably and agreeably, in enlarging our store of literary and professional knowledge; and I trust you will not allow these golden moments to escape unimproved. For while it will be acknowledged that you enter the profession much better qualified to practice it skilfully than were many of the veterans who are nearly ready to leave their places to you, yet you can not retain your present position, unless you *continue* your studies. You must constantly advance, and rapidly too, or you will soon find yourselves in the rear instead of the van of the profession. You have here been taught many important principles, and the manner in which they should be applied in general practice; but you will now be required to correlate these principles and to apply them in the various and intricate details of every-day practice. In the words of the inexorable policeman in Bleak House, to poor Jo, you must *move on*; and the progressive spirit of our profession will ever continue the refrain, " move on, move on;" until the science and art of dentistry have reached the *Ultima Thule* of perfection. While the profession is thus advancing, the broad principles which you have been taught here, will remain solid and immovable. New truths will, of course, be constantly unfolded; and the lectures delivered from year to year in this Institution will be so modified as to embrace them.

Should you return a few years hence to this beautiful city—this " Paris of America "—you will find that though its principal streets, with their grand edifices, remain without observable change, yet you will discover new avenues here and there; and your eyes will be delighted with the sight of many a stately mansion, adorning the sites that are now vacant, or covered with miserable shanties. So too, if you should then visit your *Alma Mater*, you will recognize the same professors, I should hope, that you now see around you; and in listening again to their voices you will find that they still hold fast to the many principles and modes of practice that have survived the tests of scientific examination and experience; but now and then you will perceive new and glittering truths falling from their lips, evincing that they too have been " moving on." You will, in fact, find that new avenues have been opened up in the domains of our science, as well as in the precincts of this fair city. Each successive graduating class then, will have ascended a degree higher, than that which preceded it, in the scale of professional education. But if you possess the ambition and the grit that I am confident you do, you will not let them pass you. As you have already got the start of all your successors, *and the inside track*, I trust you will never take their dust, as you surely will do, if you remain satisfied with your present attainments. In fine, let your answer be that of Plato, when asked the question; "How long do you intend to pursue your studies?" "As long," said he "as I am not ashamed to grow wiser and better."

The present epoch "booms" with unsurpassed physical and intellectual activity. Inventions in the arts, discoveries in the sciences, follow each other so closely that they must march like soldiers, "in close order," to avoid treading upon each other's heels. The elements have been subjugated, and are become the safe and obedient servant of man. Not long ago, Ulysses—so Homer tells us—had all the winds (except Zephyrus) bound and imprisoned. In fact he had literally *bagged* some of the most powerful elements of nature. This control of the subtile forces, Ulysses considered a great triumph, a grand *coup d'etat*, or rather *coup des vents*. But, though he held them in brief captivity, he could not command their services; they soon escaped, and to himself and his companions they became masters, instead of slaves. But now the mighty monarch of Olympus, "the cloud compelling Jove," has been robbed, not only of his winds, but of his *lightening*, which has been tamed and civilized, and become the useful servant of mortals. It has not only been made a fleet errand boy, carrying messages in the twinkling of an eye to the remote corners of the earth, but it has driven black night from our dwellings and our streets. It has been taught to *speak* in every tongue, and to *write* in every language, and has recently become an accomplished musician. Its harmonious notes glide along the tuneful wire, to burst upon the far distant ear, and fill it with rapturous melodies. The achievements of this wondrous force can not be enumerated here, nor can its capabilities be estimated or even imagined. The equally marvelous, nor less useful, *domestic* servant, the Telephone, has also come into popular favor almost in a day, lessening the labors and adding to the convenience of man. The Phonograph too—the tell-tale tin-foil—excites our utmost wonder; mimics our voices, and repeats our words with startling exactness. Even the teeth, which were long regarded as rather the enemies of sight and hearing, and which have sometimes been torn from the jaws of both man and beast, for real or imaginary offences against the organs of these senses, have now assumed the functions of one of these important organs; and many, who never had heard the voice of man or of nature, now rejoice in the discovery of this blessed avenue of sound. Indeed the orator of to-day might appropriately appeal to some of his auditors to lend him their *teeth* instead of their *ears.* These are only a few examples of the discoveries resulting from the mental energy and activity of the present day. The entire evening might be spent in simply enumerating them.

And the profession of Dentistry, though its progress is not marked by any very *startling* discoveries, either in the art or the science, is nevertheless advancing with great rapidity; and is fast becoming entitled—if not to a high rank among the *learned* professions—at least to a reputable position among the *healing arts.*

As a whole our profession is advancing; but the *individual members* march with unequal pace, and often *pursue different routes.* In climbing the hill of science only a few of the most vigorous and daring are able to take the most direct course; the great multitude can only ascend, as one climbs the Alpine heights, by zigzags, or tortuous paths. The collective mass however moves; though many individuals block up the paths by their inertia, and only advance, by being elbowed on by the crowd that passes them. These are the *impedimenta* of the profession, which are pressed forward little by little by the jostling, irresistible throng. They resemble barnacles on the drifting log; the wind and tide carry them along, but they never "paddle their own canoe." Like the inhabitants of a certain fabled island, they move because the island *drifts.* But their progress is slow and uncertain, and sometimes backward; the sooner such men drop out, the better it will be for the profession and for the world.

Although much has already been accomplished by special legislation in several of our states, for the gradual extermination of these unqualified practitioners; yet it seems to me that much good might result, both to the public and the competent practitioner if we should put in practice, some of the criminal codes enacted by the ancients with such modifications as our exigencies require. For instance, Diodorus tells us that Actisanes, King of Æthiopia, a ruler "who was remarkable for his moderation toward his subjects, as well as for his justice and equity," had all the robbers and malefactors collected from every part of his Kingdom, and having had their noses cut off, sent them to a city which was founded for them, called Rhinocolura. Now if our government would follow the example of Actisanes, and establish a city for the exclusive habitation of these professional robbers of the teeth, it would be a quick, and it seems to me, appropriate mode of disposing of such malefactors. For this city, *Edentata* would be as apt a name as *Rhinocolura, or Cut-nose* was for the former; since the entire population would very soon become edentulous, if the citizens continued to follow their old trade and to practice it upon each other.

I do not anticipate that any of you gentlemen would be found among the inhabitants of this city. The energy and ambition which have carried you triumphantly through your college course will not wither and die, as soon as you leave the bracing atmosphere that pervades your *Alma Mater*. But we cannot forget the painful fact, that a few have borne away from our colleges the same sacred parchment, and yet have, by their unprofessional conduct, hindered rather than aided the progress of Dental Science; who have, in fact, disgraced, rather than honored their *Alma Mater* and adopted profession. Again, there are a few who have hidden their light under a bushel; who have not only thrown no light upon the profession, but, what is worse—for them at least—they have hidden themselves under the same bushel so that no light from without could reach them.

Dentistry is in many respects an experimental science; and its best modes of practice can be determined only by comparison. In order to approach more nearly to perfection, your operations and researches, and the results of your observations, must be examined and *compared* with those of your fellow practitioners. This can be done in the meetings of our Associations, better than any where else. In these, it must be confessed, you will be compelled to listen to much of unprofitable discussion, much of unsound theory and absured practice; but by carefully examining the mass of material that is laid before you, you cannot fail to find something that may be made available. And though you may be astonished at the many and diverse modes of practice that are advocated, each of which is the only true one, in the estimate of its champion, you must remember that, according to the old adage, "all roads lead to Rome." So, in the practice of Dentistry, though all roads are not equally good or direct, yet many of them lead to the same or practically the same results.

Besides, the stimulating influence of our Associations is infectious. As one musical instrument catches the sound from another of the same pitch, so does the dentist catch new ideas and fresh inspiration from associating with his fellow practitioners. If two clocks, having pendulums of the same period of vibration, be placed in favorable positions, and one of the clocks be set agoing, the ticks of the running clock will soon start its neighbor to running. So the mingling of men of like professions and kindred spirit will awaken the genius that may be dormant within them, and render active the potentialities that might remain forever inert and useless.

And now gentlemen, I hope you will pardon the remark I am about to make; it may be because you are surrounded by so many giants, but really in spite of your manly proportions and mental acquisitions, I can only look upon you as *babes;* not the common article, but rather like such as were sometimes born in the olden time—the precocious Mercury, for instance, who you may remember, slipped from his cradle the night after his birth, while his mother slept, and stole a yoke of oxen from Admetus. Not that I would impute to you the moral qualities of that light-fingered infant, for you only resemble him in being remarkably smart babies, and evidently able to cope with the old folks. Indeed, as I look upon you, full of ambition and aspirations for fame, I am forcibly reminded of a passage in one of the dramas of Plautus, in which Stratophenes, a captain in the Babylonian army, on being told that he had just become the father of a son, inquires; Is it like me? The nurse replies: "Like you indeed! why, the very moment it was born it called for a sabre and shield!'

The proud parent then exclaims: "And has it already chosen some army that it intends to plunder?"

Now though the *profession* into which you have just been born, bears no resemblance to that of the young Stratophenes, yet the intense eagerness manifested by this baby-hero to commence business on his own *hook*, is, I doubt not, common to you all; and your anxiety is mainly concentrated upon the important question: "How am I to get a start in business?"

I shall not attempt to answer this question, but will briefly touch upon a few points, which seem to me to be essential. You are about to grapple in earnest with all the realities and responsibilities that attend the practice of a useful and beneficent profession. Armed with the implements of your calling, and supplied with a considerable stock of practical and theoretical knowledge of its duties, you issue from your *Alma Mater*, not unlike Minerva from the brain of Jupiter, armed and equipped for the great battle of life. You go forth with young, stout hearts to occupy different fields of labor; but in whatsoever direction you may go, you will find laborers there before you; some preparing the stubborn soil, some sowing the seed and some reaping the harvest. But do'nt flatter yourselves, gentlemen, that your only business will be to garner the golden grain; neither let this prospect discourage or dishearten you, for you commence your career under far more favorable auspices than the most of these pioneers did, and you will find far less obstacles to encounter than they have overcome. Besides, the experimental knowledge which they have gained, is not kept secreted with the watch-dog care that it once was, but it is imparted with a liberality and generosity equal to the niggardly secrecy of former days. While this is true, still your own persevering and determined efforts, properly directed, and the skillful and conscientious treatment of the cases intrusted to your care, as well as your courteous and gentlemanly bearing must all be made auxiliary to the title you have earned here, before you can receive the full respect and confidence that have already been accorded to a few of the eminent practitioners now in the field. Your Diplomas will be regarded as *prima facie* evidence of your competency as a practitioner, and will give you, and justly by so, considerable advantage over those who have entered the profession without such guarantee of qualifications. The simple fact that you have graduated at a reputable Dental School, will give you, at the very outset, an honorable position among your professional brethren, and a favorable place in public esteem. Of course you must form acquaintances in various proper and legitimate ways, and your position in society and the character of your patrons will depend largely upon that of the associates you make and by whom you are introduced. If your tastes and education are such as to fit you for the enjoyment of the most refined and cultured circles of society, you will naturally find your home among these, though your practice may extend beyond the bounds of this circle. But, as a rule, your practice, if satisfactory, will spread from family to family, among those who are more or less connected by the ties of friendship and congeniality of taste. In addition to your educational accomplishments and gentlemanly bearing, you must prove yourself to be a skillful and scientific dentist. Whatever you may find to do, let it be done in the best possible manner; for on the *perfection* of your work you must mainly rely for securing and retaining a good practice. Study carefully the character and nature of the disease you are about to treat; the therapeutical remedies to be applied, or surgical operation to be performed. If you have cases in what is called Mechanical Dentistry, and I trust you have not been taught to regard this essential, but much abused branch, as beneath your professional dignity, for no manual operation in the mouth requires a higher degree of purely artistic skill than may be displayed in this; if, I say, you have cases in this branch, adopt the suggestions outlined in the "Primer" on the *Mouth and the Teeth*, by Dr. J. W. White; and your skill will soon be appreciated and rewarded. Should you disregard this branch of dentistry, you would slight what may be made a source of profit, and at the same time afford a necessary relief from monotony.

Without particularizing farther, I would urge upon you the importance of doing all you undertake with the greatest care and the strictest fidelity. Upon this point you will find in the homely doggerel sung by Sir Joseph, K. C. B., a weighty suggestion and one that may be made available and profitable to you all:—

"And I polished up that handle so carefullee
That now I'm the ruler of the Queen's Navee."

And now, gentlemen of the graduating class, allow me to congratulate you upon the successful termination of your collegiate studies and the bright prospect that lies before you. You are about to separate from many friends who have become dear to you, and to leave scenes and objects that will not soon cease to interest you; but the events of this day, which are little heeded, perhaps, outside of a small circle of friends, are monumental events which can never be obliterated from your memory. However full of incident may have been your past, and however eventful may be your future career, to-day is the era from which you will date your life's history. No pyramid of Cheops or Chephren ever stood more firm or enduring than the monumental shaft that these events have just completed. It will never disappear, like the retreating, fading shores, as you sail out on your returnless voyage; but as the rolling years widen the distance between it and you, it will be lifted higher and higher above the horizon by the mental mirage, *increasing* rather than diminishing in beauty and splendor. The features of your classmates, and especially of your teachers, will be indelibly chiseled upon its imperishable surface, and many a golden line will perpetuate the principles which you have here been taught. Even those precepts which you may have only partially grasped or partially appreciated here, will assume a clearer aspect, and your bosoms will often be made to swell with gratitude to the Faculty of this Institution, who have so arduously and zealously toiled in your behalf.

But gentlemen, the monumental column to which this day's events add the crowning stone, belongs, not only to each of you separately, but to all of you in common; each individual, then, should be ever watchful that no unprofessional act of his shall mar its pure and polished surface or dim its lusture. A dishonorable act of any one of you, would not only injure yourself, but, just as the derangement of a single function of the body, disorders the whole system, so, in a measure, would such an act cast a stigma upon the entire class, upon the Institution you now leave, and upon the profession you are about to enter.

Since the Institution has lifted you to a higher plain than that you occupied when you entered it, may I not with propriety, remind you gentlemen of your obligation to lift it to a higher position. This you can easily accomplish by the honest work you shall do, by the honorable character that you shall bear, and by the influence that you shall exert in its favor; and still further, by the gifts some of you will be able to bestow upon it. This college is your *Alma Mater*, your cherishing mother; cherish her as she has cherished you. With smothered feelings of love and anxiety, she now, like the Spartan mother, hands you your armor and your weapons, and with trembling lips bids you fight manfully and gloriously the battle of life; *and either bring back safely the shield, or be brought back upon it.* And this sentiment finds an echoing voice in the hearts of us all.

